# Risk stratification and prognosis prediction based on inflammation‐related gene signature in lung squamous carcinoma

**DOI:** 10.1002/cam4.5190

**Published:** 2022-09-03

**Authors:** Wenyu Zhai, Si Chen, Fangfang Duan, Junye Wang, Zerui Zhao, Yaobin Lin, Bingyu Rao, Yizhi Wang, Lie Zheng, Hao Long

**Affiliations:** ^1^ Department of Thoracic Surgery Sun Yat‐Sen University Cancer Center, Collaborative Innovation Center for Cancer Medicine, State Key Laboratory of Oncology in Southern China Guangzhou China; ^2^ Lung Cancer Research Center Sun Yat‐Sen University Guangzhou China; ^3^ Department of Medical Oncology Sun Yat‐Sen University Cancer Center, Collaborative Innovation Center for Cancer Medicine, State Key Laboratory of Oncology in Southern China Guangzhou China; ^4^ Medical Imaging Division, Department of Medical Imaging and Interventional Radiology Sun Yat‐Sen University Cancer Center, Collaborative Innovation Center for Cancer Medicine, State Key Laboratory of Oncology in Southern China Guangzhou China

**Keywords:** immune infiltrated landscape, inflammation‐related genes, lung squamous carcinoma, prognostic model, risk stratification

## Abstract

**Background:**

Inflammation is known to have an intricate relationship with tumorigenesis and tumor progression while it is also closely related to tumor immune microenvironment. Whereas the role of inflammation‐related genes (IRGs) in lung squamous carcinoma (LUSC) is barely understood. Herein, we recognized IRGs associated with overall survival (OS), built an IRGs signature for risk stratification and explored the impact of IRGs on immune infiltration landscape of LUSC patients.

**Methods:**

The RNA‐sequencing and clinicopathological data of LUSC patients were downloaded from The Cancer Genome Atlas (TCGA) and the Gene Expression Omnibus (GEO) database, which were defined as training and validation cohorts. Cox regression and least absolute shrinkage and selection operator analyses were performed to build an IRG signature. CIBERSORT, microenvironment cell populations‐counter and tumor immune dysfunction and rejection (TIDE) algorithm were used to perform immune infiltration analysis.

**Results:**

A two‐IRG signature consisting of KLF6 and SGMS2 was identified according to the training set, which could categorize patients into two different risk groups with distinct OS. Patients in the low‐risk group had more anti‐tumor immune cells infiltrated while patient with high‐risk had lower TIDE score and higher levels of immune checkpoint molecules expressed. The IRG signature was further identified as an independent prognostic factor of OS. Subsequently, a prognostic nomogram including IRG signature, age, and cancer stage was constructed for predicting individualized OS, whose concordance index values were 0.610 (95% CI: 0.568–0.651) in the training set and 0.652 (95% CI: 0.580–0.724) in validation set. Time‐dependent receiver operator characteristic curves revealed that the nomogram had higher prediction accuracy compared with the traditional tumor stage alone.

**Conclusion:**

The IRG signature was a predictor for patients with LUSC and might serve as a potential indicator of the efficacy of immunotherapy. The nomogram based on the IRG signature showed a relatively good predictive performance in survival.

## Graphical abstract

Based on a variety of bioinformatics and immune infiltration analyses, we found the expression of inflammation‐related genes was associated with the prognosis. And the inflammation‐related genes signature can be a potential indicator of the effectiveness of immunotherapy.

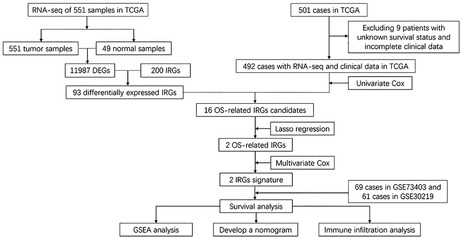

## 1 INTRODUCTION

Lung cancer, the second common malignant tumor, leads to the most cancer‐related deaths around the world,^1^ among which, lung squamous carcinoma (LUSC) is the second most common histological types, which accounts for approximately 25%–30% of all lung cancers.^2^, ^3^ Despite great improvements in therapy in recent decades, the survival of LUSC patients remain poor, especially in those without driver gene mutations.^4^, ^5^ Though tumor‐node‐metastasis (TNM) system is widely applicated in clinic, its predictive performance is limited due to neglecting genetic characteristics.^6^ Hence, it is very meaningful to explore novel prognostic indicators and construct a prognostic model as a supplement to traditional TNM system, which can better predict survival and guide personalized therapy for patients with LUSC.

Inflammatory response plays a critical role in infection, trauma, and other stresses.^7^ Inflammation is also known to have a strong relationship with tumorigenesis.^8^, ^9^ Studies showed that aberrant inflammation response and status could contribute to lung cancer.^10^, ^11^ Recent studies have revealed that prognosis in patients with colon cancer can be predicted by combining analysis of local infiltration of chronic inflammatory cells and systemic inflammatory responses.^12^ The systemic immune‐inflammation (SII) index has been explored as a prognostic‐related indicator in multiple cancers, high SII index is related to a poor prognosis in non‐small cell lung cancer (NSCLC).^13^ Generally speaking, the relationship between cancer‐related inflammation and cancer works in two different ways, including local and systemic inflammations.^8^ Local inflammation is considered to reflect the local immune response, including the tumor‐and host‐derived cytokines, immune cells acting, and inflammatory proteins on the tumor immune microenvironment (TIME).^14^ Systemic inflammation includes circulating immune cells and acute‐phase proteins. Reactive oxygen species are generated via the inflammatory processes, and they promote tumor generation by inducing deoxyribonucleic acid (DNA) damage and mutations.^9^, ^15^ In the TIME, more distinct proinflammatory cytokines, such as interleukin (IL)17,^16^ IL32,^17^ and IL6^18^ are secreted compared to the tumor‐free tissue. Changes in neutrophil and lymphocyte numbers and their ratio may affect the survival in lung cancer.^19^ C‐reactive protein closely participates in the oncogenesis derived from chronic pulmonary inflammation.^20^ Nevertheless, the clinical value of inflammation‐related genes (IRGs) in LUSC is poorly elucidated.

In current study, the expression pattern and clinical value of IRGs were investigated using the expression profile datasets of LUSC patients obtained from The Cancer Genome Atlas (TCGA) LUSC project. We established an IRG signature for predicting individualized OS of LUSC patients, and also tested its predictive value via date from other two independent Gene Expression Omnibus (GEO) dataset. In addition, the expression difference of IRGs between normal and tumor tissues was assess using immunohistochemistry (IHC) in formalin‐fixed, paraffin‐embedded (FFPE) tissue sections. Finally, we combined the IRG‐based scores and TNM stage of LUSC to develop a nomogram for precisely predicting individualized patients' outcomes.

## 2 MATERIALS AND METHODS

### 2.1 Data acquisition and preparation

The count data of RNA‐sequencing profile and clinical data of LUSC patients were acquired from the TCGA databases, and the count data were conducted log_2_ transformed before further analysis. We also obtained the gene expression array from anther two GEO databases (GSE73403 and GSE30219), which were also log_2_ transformed. After excluding cases with absence of clinical information and those lost to follow‐up, 492 LUSC cases obtained from the TCGA comprised the training set, and 130 cases (69 cases of GSE73403 and 61 cases of GSE30219) comprised the validation cohort. The last process of data preparation was removing the batch effect from above datasets via the “limma” and “SVA” R packages. And 200 IRGs were identified from the Molecular Signatures Database.

### 2.2 Construction and validation of the IRG signature

The differentially expressed genes (DEGs) between LUSC tumor and normal lung tissues were decided by the “limma” R package with log_2_| fold change | >1 and false discovery rate (FDR) <0.05. The genes at the intersection of DEGs and IRGs were identified as prognostic IRG candidates. Then, univariate Cox and least absolute shrinkage and selection operator (LASSO) regression analyses were further performed in the training cohort to identify final IRGs. Subsequently, the IRG risk score of patients using the normalized count of final IRGs with their corresponding coefficients from multivariate Cox regression model. The specific formula of risk score = sum (IRGs' expression level × corresponding coefficients).

### 2.3 Functional annotation and pathway enrichment analyses

To elucidate the potential pathways enriched in identified IRGs for LUSC patients, we performed the Gene Set Enrichment Analysis (GSEA) in high‐risk groups in the TCGA training set. The GSEA 4.0.1 software was utilized to perform Genetic Ontology (GO) term and Kyoto Encyclopedia of Genes and Genomes (KEGG) path analyses. After 1000 permutations, significant enrichment was defined as the pathway with the value of FDR < 0.25 and normalized *p* < 0.05.

### 2.4 Immune infiltration and tumor mutation burden (TMB) analyses

To assess 28 kinds of immune infiltrating cells, we performed the single sample GSEA (ssGSEA) using the “GSVA” package.^21^ CIBERSORT algorithm is widely used for analysis characters of 22 immune cells infiltrating in the TIME using the gene expression profile. With the perm set to 1000, characters of 22 immune cells were calculated.^22^ The Masked Somatic Mutation data were acquired from TCGA via the “maftools” R package.^23^ Besides, another algorithm, microenvironment cell populations‐counter (MCP‐counter) was also performed to estimate abundance of 10 types of cells between high and low‐risk patients.^24^ Tumor immune dysfunction and rejection (TIDE) scores were acquired to assess the treatment benefit from PD‐1 inhibitors.^25^ The TMB calculation is equal to the total mutation frequency/35 MB. Therefore, TMB per megabase was obtained by dividing the total number of mutations by the size of the coding region of the target. The expression levels of 4 immune checkpoint molecules (ICMs), PD‐1, PD‐L1, PD‐L2, and CTLA4 and TMB between two risk groups were compared with the Mann–Whitney U test.

### 2.5 Establishment of a prognostic nomogram

In the TCGA cohort, a prognostic nomogram for predicting individualized OS was developed while calibration curves of OS at 1‐, 3‐, and 5‐year were plotted both in the TCGA training and the GEO validation sets to test the predictive performance of this prognostic nomogram. Time‐dependent receiver operator characteristic (ROC) curves were performed to compare the prognostic accuracy between our nomogram and cancer stage alone.

### 2.6 Verification of the protein expression using IHC

We collected nine pairs of LUSC tumor tissue and adjacent normal tissue samples from January, 2016 to January, 2017 at the Sun Yat‐Sen university cancer center. The FFPE tissue sections were dewaxed and rehydrated, and subjected to antigen retrieval and blocking. First of all, the slides were incubated with anti‐rabbit primary antibodies overnight at 4°C. The primary antibodies included anti‐KLF6 polyclonal antibody (1:1000; ab241385; ABCAM) and anti‐SGMS2 polyclonal antibody (1:1000; ab237681; ABCAM). Then, after incubation with HRP‐conjugated rabbit polymer (1:500; ab97051; ABCAM), the secondary antibody, we used liquid diaminobenzidine tetrahydrochloride, and substrate (DAB chromogen, Changjiang) followed by counterstaining with hematoxylin for visualization. Finally, a light microscope (Nikon) was used to photograph the samples, which were analyzed using ImageJ (FIJI v2.1.0). A semiquantitative scoring system was conducted to assess and score the sample ranging from 1 (minimum) to 4 (maximum).

### 2.7 Statistical analysis

Categorical variables were shown as the rate and continuous data were displayed as the mean ± SD. To determine the cut‐off value of the risk score, maximally selected log‐rank test was performed via the “maxstat” package for patients' stratification. Survival curves were calculated using the Kaplan–Meier method and compared using the log‐rank test. The multivariate Cox model was conducted to determine the significantly independent indicators of OS for patients with LUSC. Subsequently, a prognostic nomogram for individualized prediction in OS for LUSC patients was developed based on the TCGA training set via the “rms” package. The predictive performance and accuracy were evaluated using time‐dependent ROC, calibration curves, and concordance index (C‐index) both in the TCGA training and GEO validation datasets. We used R software (version 4.0.1) to perform all statistical analyses. Statistical significance was considered as *p*‐value <0.05.

## 3 RESULTS

### 3.1 Identifying the IRG signature

**FIGURE 1:**
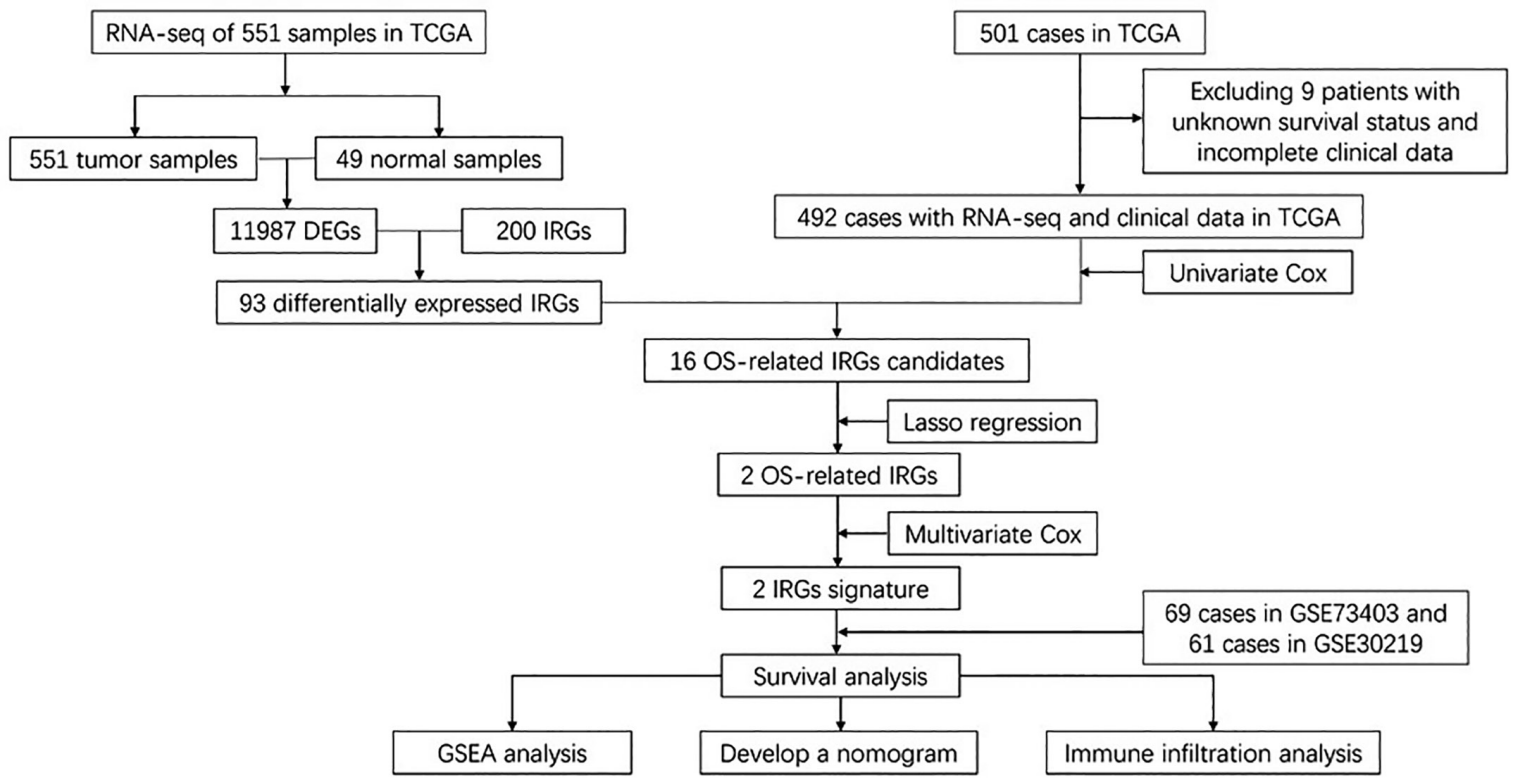
Flow chart of this study

DEGs 11,987 were screened using the differential expression analysis across 551 LUSC tumor and 49 normal samples from the TCGA database. After taking the intersection of the screened DEGs and 200 IRGs, 93 genes were identified as differential expressed IRGs. After excluding nine patients with unknown survival statuses and incomplete clinical information, there are 492 cases from the TCGA database comprising the training cohort. The GEO validation cohort comprised 130 cases, which included 69 cases of GSE73403 and 61 cases of GSE30219 (Figure 1). Table 1 showed the clinical factors of TCGA training and GEO validation sets.

**TABLE 1:** Patients' characteristics

	TCGA training cohort *n* = 492	GEO validation cohort *n* = 130
Gender		
Male	364 (74.0)	121 (93.1)
Female	128 (26.0)	9 (6.9)
Age (year)	61.3 ± 9.6	58.3 ± 8.5
≤65	171 (34.8)	79 (60.8)
>65	321 (65.2)	51 (39.2)
Smoking history		
No	18 (3.7)	11 (8.5)
Yes or ever	474 (3.7)	58 (44.6)
Unknown	0	61 (46.9)
T stage		
T1	114 (23.2)	53 (40.8)
T2	286 (58.1)	47 (36.2)
T3	69 (14.0)	25 (19.2)
T4	23 (4.7)	5 (3.8)
*N* stage		
N0	320 (65.0)	86 (66.2)
N1	127 (25.8)	27 (20.8)
N2	40 (8.1)	17 (13.2)
N3	5 (1.0)	0 (0)
M stage		
M0	486 (98.8)	130 (100)
M1	6 (1.2)	0 (0)

**FIGURE 2:**
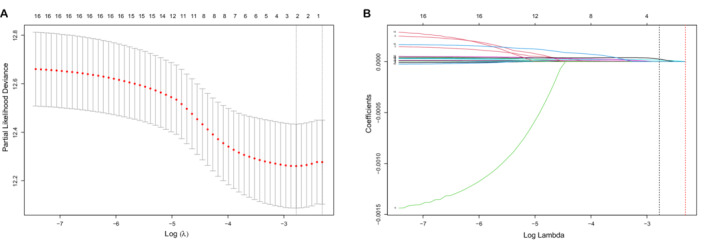
Lasso regression model in the TCGA training cohort. (A) Selection of the optimal candidate genes; (B) LASSO coefficients the IRGs

And 16 candidate prognostic‐related IRGs were identified from the univariate Cox analysis (Figure S1). Among the 16 IRGs, two genes, namely *KLF6* and *SGMS2*, were significantly associated with OS in subsequent LASSO analysis (Figures 2A,B). Finally, we established a risk score consisting of the expression levels of these two IRGs and their coefficients obtained from the multivariate Cox regression analysis (Figure S2). The formula of risk score = KLF6 × 2.875e‐5 + SGMS2 × 8.359e‐5.

### 3.2 Prognostic value of the IRG signature in the TCGA training cohort

**FIGURE 3:**
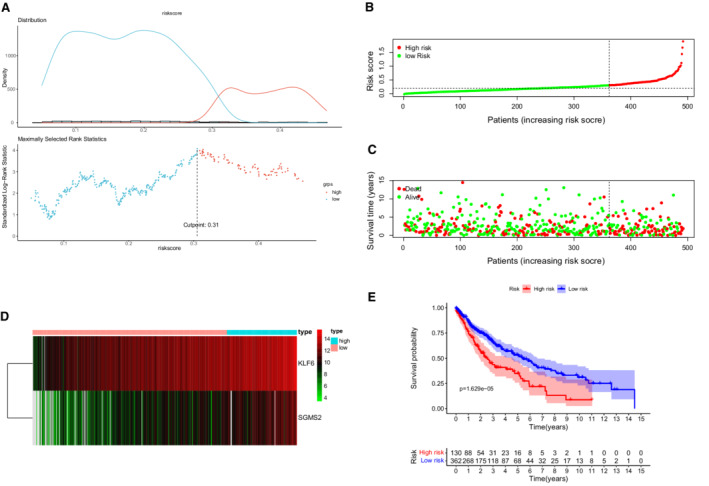
The prognostic value of the IRG signature in the TCGA training cohort. (A) The maximally selected log‐rank statistics determined cutoff value of IRGs risk scores. (B) Patient distribution in the two risk groups according to the risk scores. (C) The distribution of overall survival (OS) status in the two risk groups. (D) The expression profiles of the two IRGs by heatmap. (E) Kaplan–Meier curves for the OS of LUSC patients in the two risk groups

The maximally selected log‐rank statistics calculated 0.31 as the cut‐off value of the risk score (Figure 3A) and LUSC patients were categorized into two different risk groups in the training cohort (Figure 3B). The high‐risk group happened significantly more death events (Figure 3C). A heat map in Figure 3D revealed a significantly higher expression of *KLF6* and *SGMS2* in the high‐risk patients. Survival curves presented that there was a significant difference in OS between two risk groups (Figure 3E *p* < 0.001).

### 3.3 Prognostic value of the IRG signature in the GEO validation cohort

**FIGURE 4:**
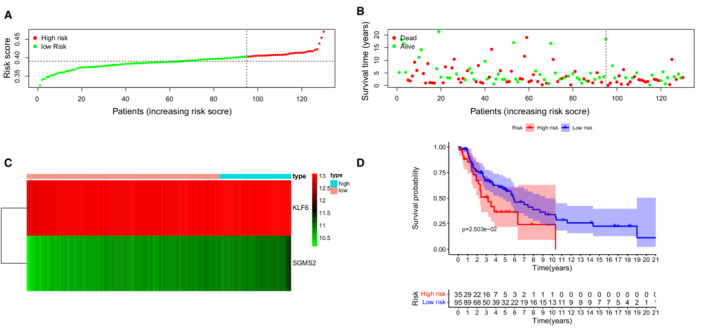
Assessment of prognostic value of the IRG signature in the GEO validation cohort. (A) Patient distribution in the two risk groups according to the risk scores. (B) The distribution of overall survival (OS) status in the two risk groups. (C) The expression profiles of the two IRGs by heatmap. (D) Kaplan–Meier curves for the OS of LUSC patients in the two risk groups.

Of the 130 cases in the GEO validation cohort, 35 and 95 LUSC patients were categorized into the high‐ and low‐risk group, respectively, according to the cut‐off value of risk score. (Figure 4A). There were more deaths in the high‐risk group (Figure 4B). The heat map in Figure 4C showed the different profiles of *KLF6* and *SGMS2* in these two groups. Survival analysis suggested a longer OS in the low‐risk group (Figure 4D *, p* = 0.025).

### 3.4 Functional annotation and pathway enrichment analyses

**FIGURE 5:**
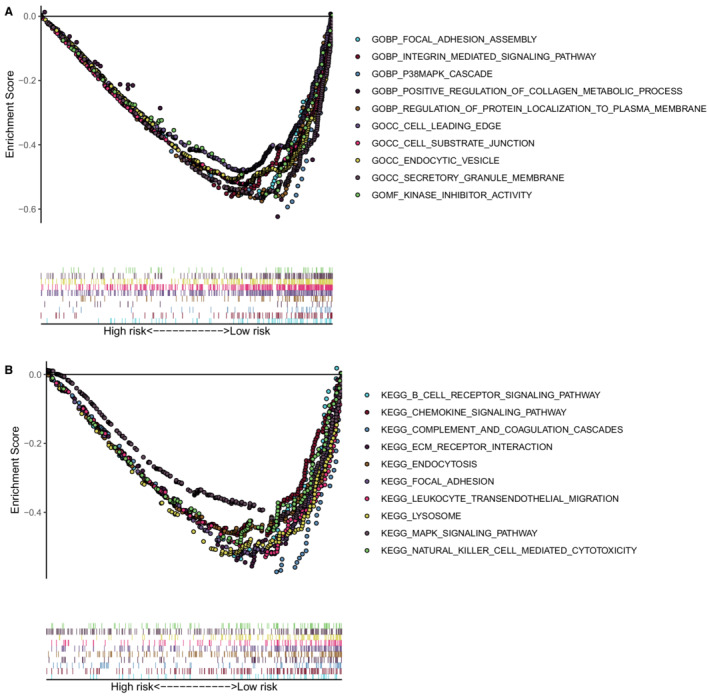
Gene set enrichment analysis (A) Enriched GO terms in high ‐risk group. (B) Enriched KEGG pathways in high‐risk group.

We performed the GO and KEGG in the training cohort to investigate the possible functional pathways enriched in the high‐risk patients. The results of GSEA demonstrated that the high‐risk group mainly enriched in focal adhesion assembly, intern‐mediated signaling pathway, p38MAPK cascade, regulation of protein localization to the plasma membrane, cell leading edge, cell‐substrate junction, endocytic vesicle, secretory granule membrane, kinase inhibitor activity, B cell receptor signaling pathway, positive regulation of the metabolic process of collagen, chemokine signaling pathway, complement and coagulation cascades, ECM receptor interaction, endocytosis, focal adhesion, leukocyte trans‐endothelial migration, lysosome, MAPK signaling pathway, and natural killer cell‐mediated cytotoxicity. (Figures 5A,B).

### 3.5 Tumor immunity landscape and TMB analyses

**FIGURE 6:**
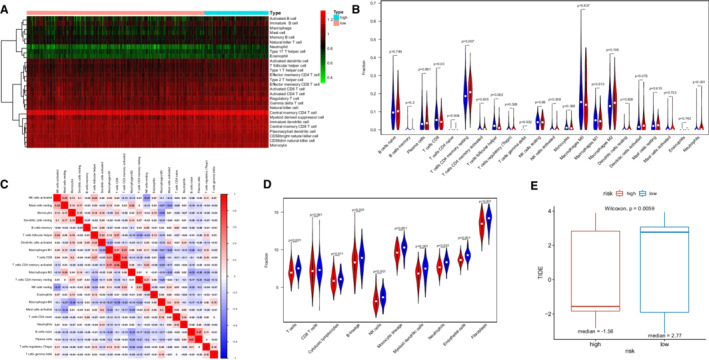
The results of immune infiltration analyses in the TCGA training cohort. (A) ssGSEA algorithm displayed the 28 tumor‐infiltrating cells proportions by heatmap. (B) CIBERSORT algorithm show the differences of 22 types of immune cell infiltrated between the two risk groups. (C) Correlation matrix of the among 22 types of immune cell infiltrated. (D) MCP‐counter show the differences of 22 types of immune cell infiltrated between the two risk groups. (E) The TIDE score of two risk groups

We used CIBERSORT and ssGSEA algorithm to investigate the potential association between IRG risk scores and tumor immune infiltration. And 28 types of immune cells infiltrated in TIME were compared between two groups using ssGSEA, which was shown in a heat map (Figure 6A). The CIBERSORT algorithm explored the infiltration levels of 22 main kinds of immune cells between the high‐ and low‐risk groups (Figure S3). Figure 6B showed that there was a higher infiltration of neutrophils, CD4 resting memory T cells, and regulatory T cells (Tregs) in the high‐risk group. We discovered CD8 T cells and follicular helper T cells infiltrated more in the low‐risk group. As shown in Figure 6C, there was a strong infiltrating correlation between CD8 T cells and M1 macrophages, CD8 T cells and CD4 memory‐activated T cells, CD4 memory‐activated T cells and M1 macrophages, naive B cells and M2 macrophages. Similar to the CIBERSORT algorithm, the MCP‐counter revealed that several kinds of anti‐tumor immune cell, such as cytotoxic lymphocytes, B cell, and natural killer (NK) cells infiltrated more in the low‐risk group. However, a higher infiltration of neutrophils was found in the low‐risk group, which is contrary to the CIBERSORT algorithm (Figure 6D).

**FIGURE 7:**
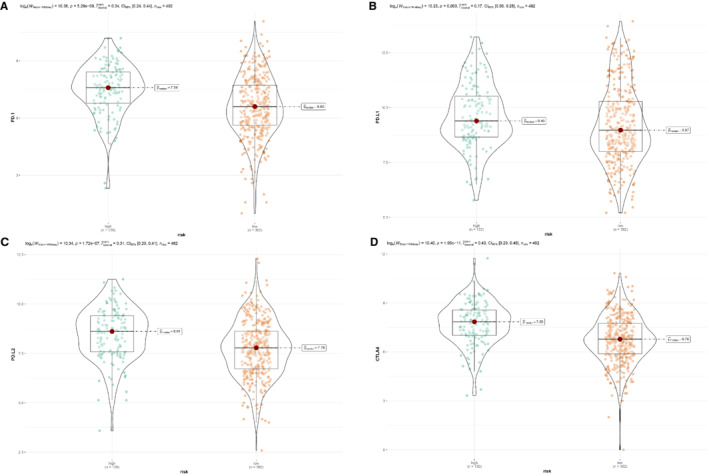
Expression of immune checkpoint molecules between the two risk groups in the TCGA training cohort. (A) PD‐1. (B) PD‐L1. (C) PD‐L2. (D) CTLA4

To investigate the value of this IRG signature in immune checkpoint inhibitors (ICIs) therapy, we compared the TIDE score between high‐ and low‐risk groups. It showed that high‐risk patients had apparently lower TIDE score, which was related to higher efficacy of PD‐1 therapy (Figure 6E). Besides, the expression levels of immune checkpoint molecules might be associated with immunotherapeutic efficacy. As we could see from Figure 7, a higher expression of PD‐1, PD‐L1, PD‐L2, and CTLA4 were seen in LUSC patients with high risk (Figures 7A–D). However, patients with LUSC in low‐risk group represented a higher TMB (Figure S4).

### 3.6 Protein expression of IRGs in tumor and normal tissues

**FIGURE 8:**
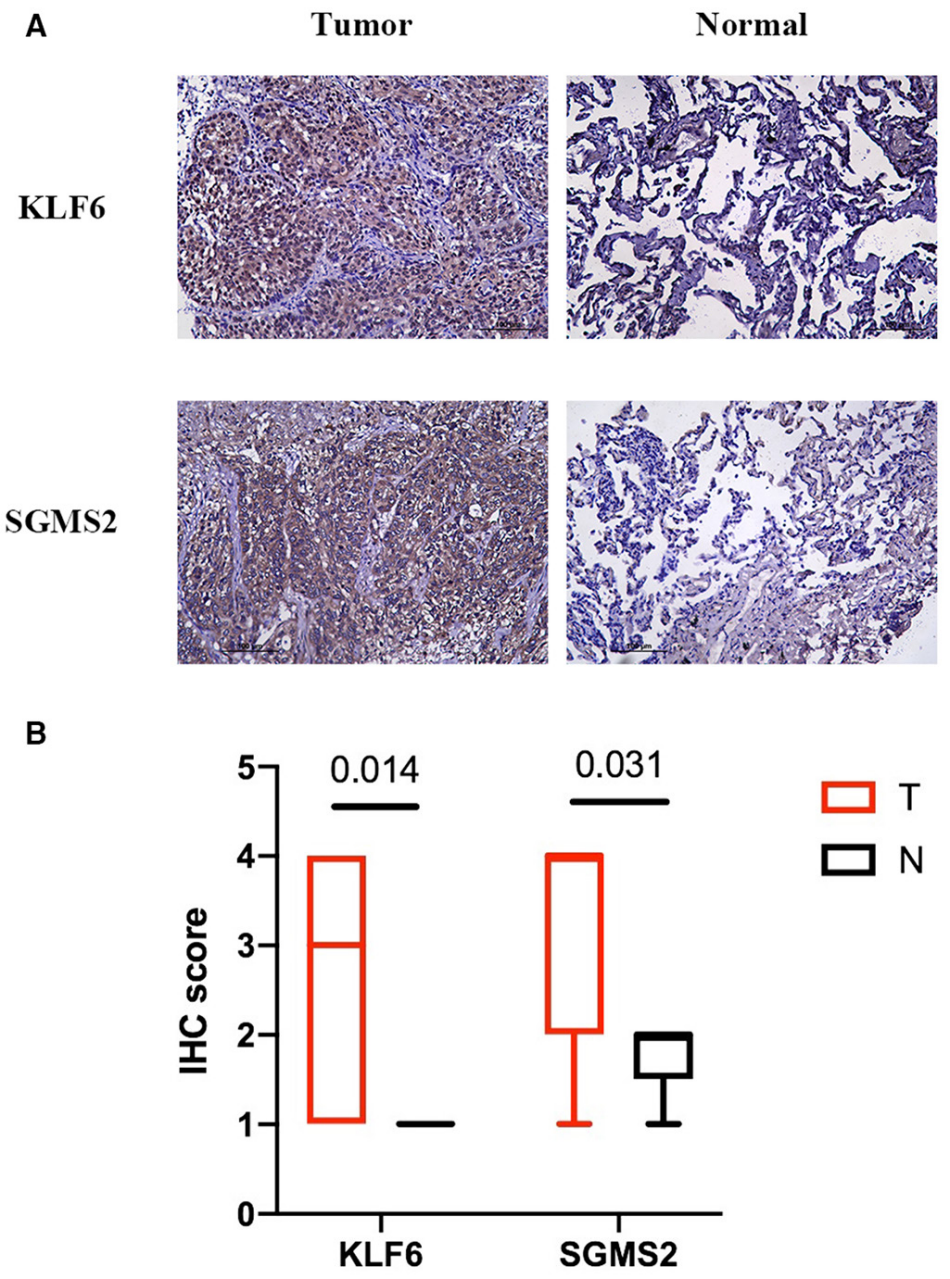
Immunohistochemical (IHC) stain of protein expression levels of 2 IRGs for LUSC tumor and para‐carcinoma tissues. (A) The representative images of IHC stain of 2 IRGs. (B) The IHC score of 2 IRGs

To determine the protein levels of *KLF6* and *SGMS2*, we obtained nine pairs of LUSC tumor and normal samples from patients for IHC staining. As shown in Figures 8A,B, *KLF6* (*p* = 0.014) and *SGMS2* (*p* = 0.031) were expressed more in tumor tissues compared to normal tissues, which suggested that *KLF6* and *SGMS2* might function as oncogenes in LUSC.

### 3.7 Nomogram based on IRG signature for LUSC

**FIGURE 9:**
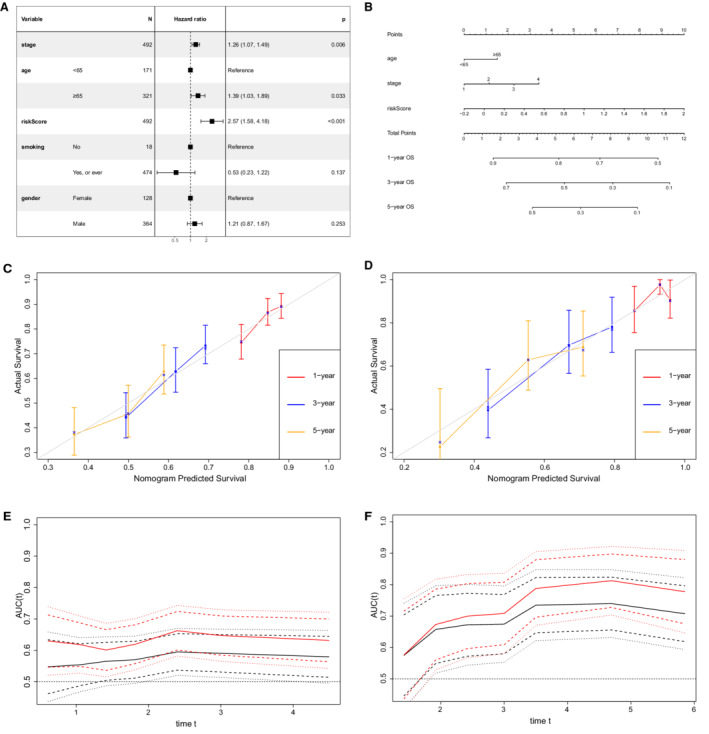
Development and validation of the prognostic nomogram. (A) Cox regression analysis. (B) nomogram model based on the stage, age, and IRG signature. (C) The calibration curves of 1‐year, 3‐year, and 5‐year OS in training cohort. (D) The calibration curves of 1‐year, 3‐year, and 5‐year OS in validation cohort. (E) Time dependent ROC curve in training cohort. (F) Time dependent ROC curve in validation cohort. Red line: nomogram; black line: stage alone

Four variables, including cancer stage, age, gender, smoking history, and the risk score, were entered in the multivariate Cox analysis, which demonstrated that age (*p* = 0.033), cancer stage (*p* = 0.006), and risk score (*p* < 0.001) were negatively associated with OS (Figure 9A). Based on these three indicators, a prognostic nomogram model was constructed to predict the1‐, 3‐, and 5‐year OS rate using TCGA training cohort (Figure 9B). The C‐index of our nomogram in the training and validation cohort were 0.610 (95% CI 0.568–0.651) and 0.652 (95% CI 0.580–0.724), respectively. The calibration curves at 1‐, 3‐, and 5‐year were drawn both in the training (Figure 9C) and validation cohorts (Figure 9D), which showed great consistency between actual and predicted survival.

Besides, the continuous time‐dependent ROC curves were plotted to compare the prediction accuracy of our nomogram and cancer stage in the TCGA training (Figure 9E) and GEO validation sets (Figure 9F), which demonstrated that our prognostic nomogram had a higher area under the curve (AUC) than the cancer stage.

## 4 DISCUSSION

The connection between inflammation and cancer is widely accepted. Several studies targeted the predictive value of inflammatory factors or immune cells and revealed that high inflammation level was correlated with poor outcomes.^26^, ^27^, ^28^, ^29^, ^30^ A previous study reviewed 2334 patients with gastric cancer and built a model using inflammatory biomarkers.^26^ Mehmet Zengin used several methods to evaluate the local inflammatory response, which is an indicator of colon cancer.^27^ Another study mentioned that a neutrophil‐to‐lymphocyte ratio is a useful serum biomarker of non‐small cell lung cancer (NSCLC).^28^ These studies mainly focused on peripheral blood cell proportions and acute‐phase protein levels. There were some studies focusing on single‐gene analysis.^31^, ^32^ Zhou and his colleagues found that mutation in the IL‐1 gene was related to a better response to immunotherapy and prolonged OS.^31^ Inhibiting BTG2 expression has been reported to increase sensitivity to radiotherapy in NSCLC.^32^ Genes work as a group, and it is necessary to analyze the inflammation‐related genes as a group. The association of transcript data of IRGs and individual prognosis in gastric cancer,^33^ hepatocellular carcinoma,^34^ lung adenocarcinoma,^35^ and colon cancer^36^ has been reported, but its prognostic value in LUSC remains poorly understood. In current study, we explored the expression pattern of IRGs and constructed an IRG signature of LUSC patients. Furthermore, we constructed a prognostic nomogram combing stage, age, and the IRG signature to predict patient outcome.

The IRG signature was based on two inflammation‐related genes ‐ *KLF6* and *SGMS2*. *KLF6* encodes a transcription factor, which is widely expressed in the nucleus and involved in cell proliferation, differentiation, apoptosis, and angiogenesis.^37^ *KLF6‐SV1* is the most common variant of *KLF6*, the overexpression of which accelerates progression and metastasis in prostate cancer^38^ and NSCLC.^39^ High level of *KLF6* was correlated with local recurrence of head and neck squamous carcinoma (HNSCC).^40^ *KLF6* expression level has also been explored to be related to local response to radiotherapy in HNSCC. In this study, *KLF6* RNA expression was negatively related to survival. IHC analysis showed that *KLF6* protein was expressed more in the tumor tissue than the normal tissue, which is consistent with RNA‐seq analysis.

The protein encoded by *SGMS2* is an enzyme that is essential to synthesize a major component of cell membranes and is indispensable for cell growth. *SGMS2* plays a role in acute lung injury and pulmonary edema^41^ and inflammatory diseases, such as diabetes mellitus and obesity.^42^ In triple negative breast cancer patients, *SGMS2* expression was related to immunosuppressive TIME and dissatisfactory survival.^43^ In the *SGMS2* knockout mouse model, the generation of M2‐type macrophages, tumor weight, and lung metastases lessened compared to the control group.^43^ It is reported that lack of *SGMS2* prolongs survival through Icam‐1 reduction in lymphoma.^42^, ^44^ Our study results were consistent.

We gained a deep understanding of the association between IRGs and the tumor immune landscape by exploring the immune infiltration in different risk patients. In both CIBERSORT and MCP‐counter analyses, we discovered some anti‐tumor immune cells, such as follicular helper T cells, CD8 T cells, cytotoxic lymphocytes, B cell, NK cells infiltrated more in the low‐risk group. CD8 T cell, the most important anti‐tumor immune cells, acts directly on cells bearing an antigen.^45^ Follicular helper T cells suppress the tumor by promoting intratumoral tertiary lymphoid structures formation.^46^ B cells play an anti‐tumor role by producing targeting antibodies.^47^ NK cells belong to innate immune cell which kill cancer cells via non‐ major histocompatibility complex ‐restrictive effects.^48^ We also found Tregs, and CD4 memory resting T cells infiltrated more in patients with high‐risk, Tregs are well‐known immunosuppressive cells, which could reduce the anti‐tumor function of other immune cells by promoting immune escape in the TIME.^49^ The difference of immune infiltration landscape was one of the possible reasons for the different survival between these two risk groups. Interestingly, we discovered that high‐risk patients might be more sensitive to immunotherapy. Firstly, patients with high‐risk had lower TIDE score, which represented a better response of PD‐1 therapy. In addition, the high‐risk group had a high ICM expression. On the one hand, a high ICM expression represents an immunosuppressed state in the primary tumor, which corresponds to a poor prognosis. On the other hand, high ICM expression shows that patients with high‐risk score may benefit more from immune checkpoint inhibitor therapy.

The current study had certain limitations. First of all, the samples were just obtained from public databases, prospective samples and multicenter data are warrant for further verification. In addition, though this prognostic nomogram presented a higher AUC than TNM stage, its predictive performance is still not very high. A real‐world cohort with more prognostic factors and transcriptome data might enhance the predictive power of IRGs prognostic model. Furthermore, the underlying mechanism of these two genes comprising the IRG signature requires more experimentation in future. The single cell RNA‐seq for the mouse model which up or down‐regulating the two IRGs may help to explore how IRGs affect the TIME. Finally, the sample size used for IHC staining to validate the protein expression of the two IRGs was limited. A more robust inference could be made with an increased number of samples.

## 5 CONCLUSION

The IRG signature we built was a valuable prognostic indicator of patients with LUSC. Patients with high‐ and low‐risk scores had distinct tumor immune landscapes. The IRG signature may serve as a potential indicator of immunotherapeutic efficacy. A prognostic nomogram integrating the IRG signature, age and stage was constructed to predict patient's OS, while its performance was better compared to the cancer stage alone. Therefore, our IRGs‐based nomogram might be used as a clinical application tool for better management of LUSC patients.

## AUTHOR CONTRIBUTION

Conception and design: Hao Long and Lie Zheng; Provision of study materials or patients: Wenyu Zhai, Si Chen and Fangfang Duan; Collection and assembly of data: Junye Wang, Zerui Zhao, Yaobin Lin, Yizhi Wang, and Bingyu Rao; Data analysis and interpretation: Wenyu Zhai, Fangfang Duan and Si Chen; Manuscript writing and editing: Wenyu Zhai and Si Chen; Final approval of manuscript: All authors.

## FUNDING INFORMATION

This study was supported by the Natural Science Foundation of Guangdong Province with Grant Numbers: 2019A1515011601.

## CONFLICT OF INTEREST

The authors declare no conflict of interest.

## ETHIC APPROVAL STATEMENT

We got approval from ethics committee of Sun Yat‐sen University Cancer Center and requirement of patient informed consent was waived. This study was conducted in accordance with the Declaration of Helsinki and personal data of patients were covered confidentially.

## Supporting information


Figure S1

Figure S2

Figure S3

Figure S4

## Data Availability

The data in this were obtained from two publicly available datasets, The Cancer Genome Atlas (https://tcga‐data.nci.nih.gov/tcga/), and the Gene Expression Omnibus (https://www.ncbi.nlm. nih.gov/geo/). 200 IRGs were identified from the Molecular Signatures Databases (http://www.gseamsigdb.org/gsea/msigdb/cards/HALLMARK_INFLAMMATORY_RESPONSE.html).
