# Supplementary figures and images for: Risk stratification and prognosis prediction based on inflammation‐related gene signature in lung squamous carcinoma
Source: Cancer Med. 2022 Sep 3;12(4):4968–80. doi: 10.1002/cam4.5190 (PMC9972108; doi:10.1002/cam4.5190)

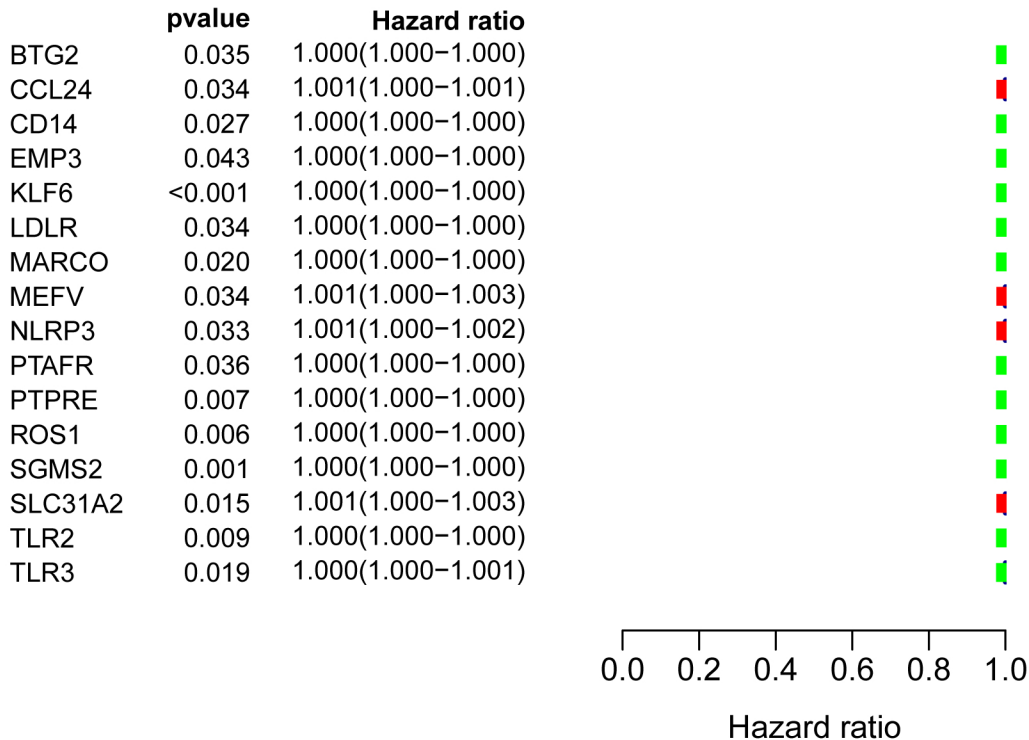

Supplement: Supplementary file 1 — Figure S1 [file CAM4-12-4968-s004.pdf]

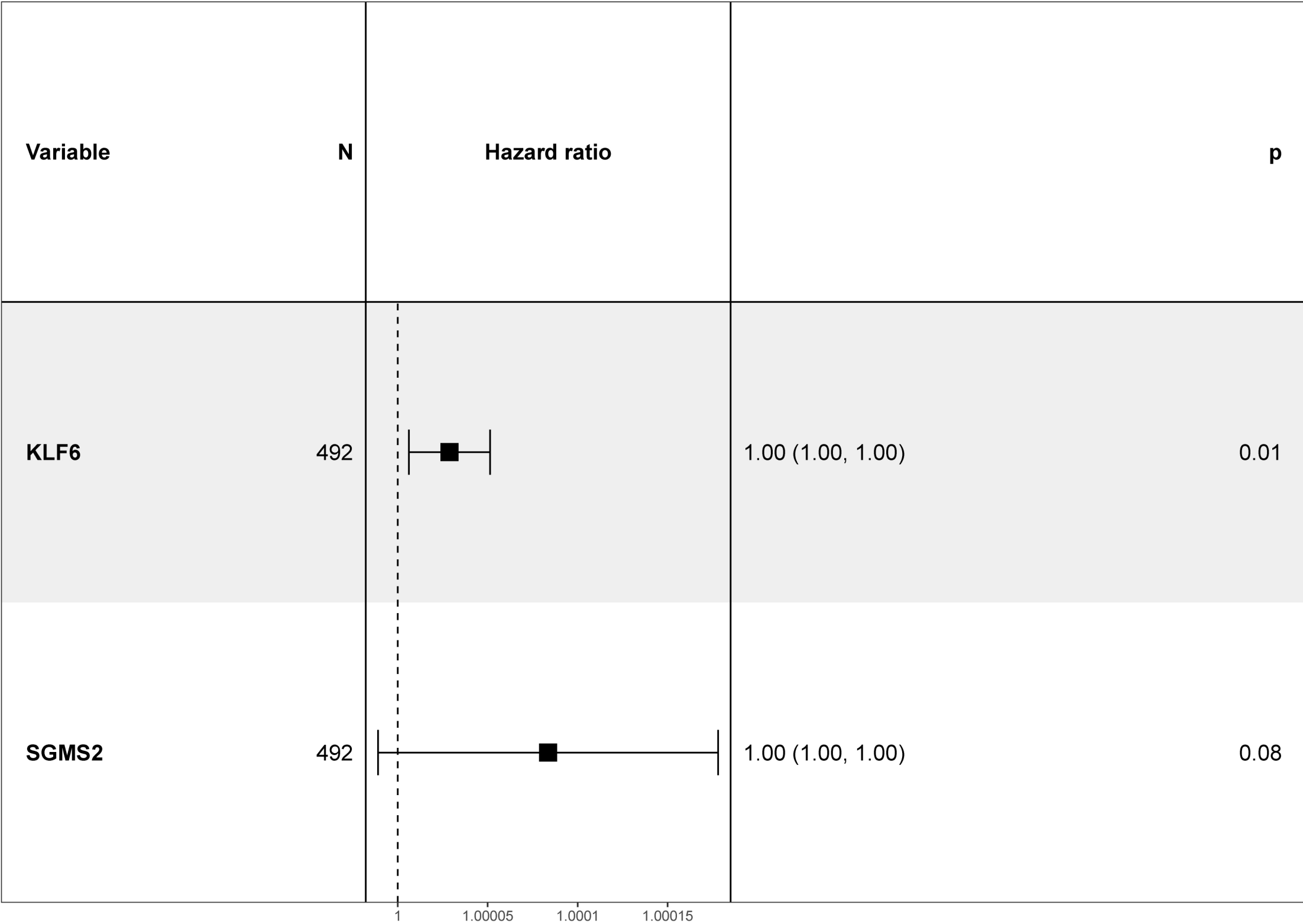

Variable

N

Hazard ratio

p

KLF6

492

1.00 (1.00, 1.00)

0.01

SGMS2

492

1.00 (1.00, 1.00)

0.08

1 1.00005 1.0001 1.00015

Supplement: Supplementary file 2 — Figure S2 [file CAM4-12-4968-s002.pdf]

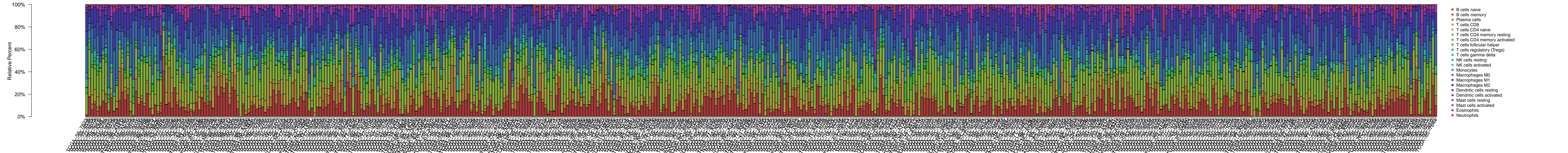

Supplement: Supplementary file 3 — Figure S3 [file CAM4-12-4968-s001.pdf]
