# Supplementary material for: Risk stratification and prognosis prediction based on inflammation‐related gene signature in lung squamous carcinoma
Source: Cancer Med. 2022 Sep 3;12(4):4968–80. doi: 10.1002/cam4.5190 (PMC9972108; doi:10.1002/cam4.5190)
Supplement: Supplementary file 4 — Figure S4 [file CAM4-12-4968-s003.pdf]

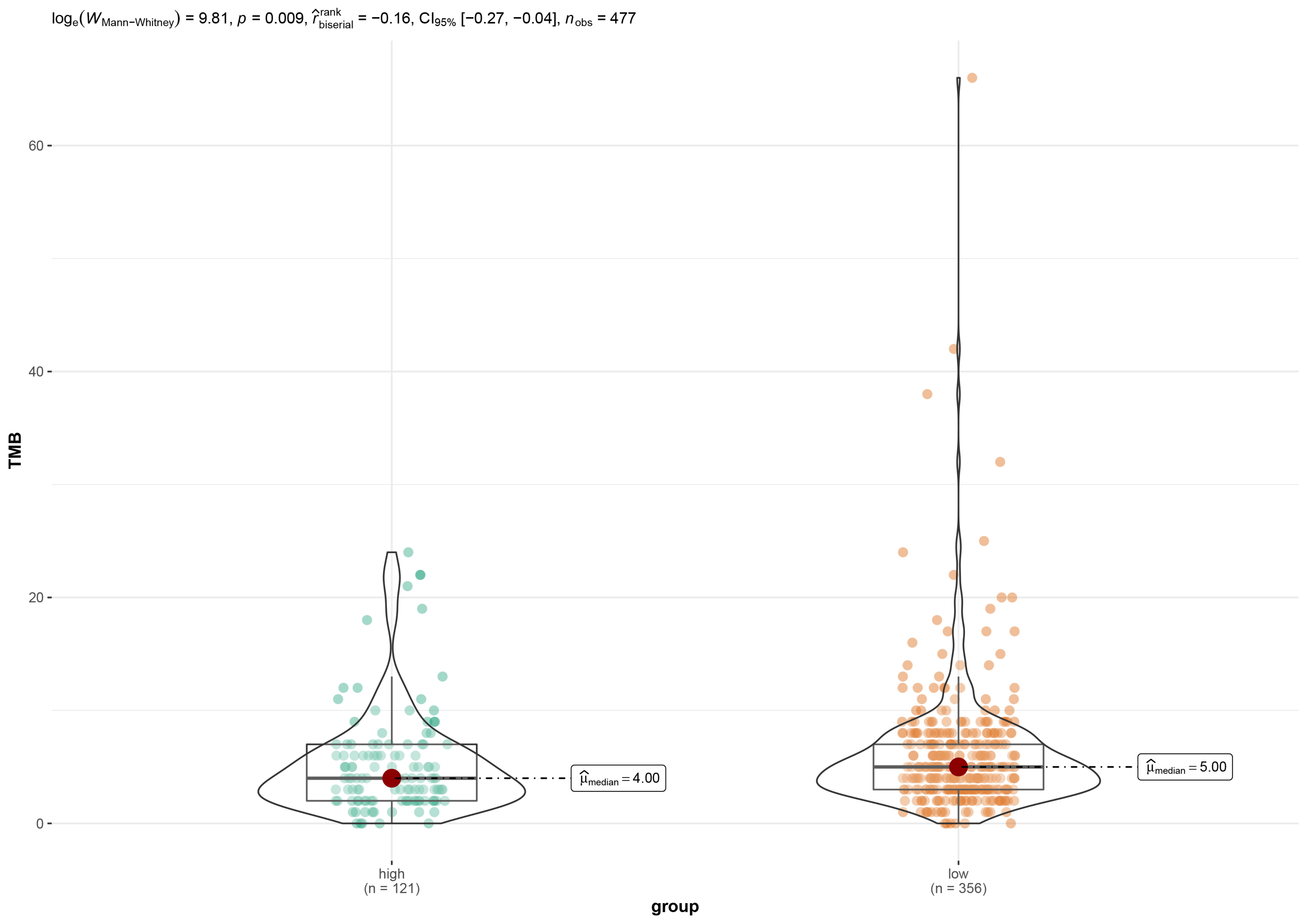
$\log_e(W_{Mann-Whitney}) = 9.81$, $p = 0.009$, $\widehat{r}_{biserial}^{rank} = -0.16$, $CI_{95\%}$ [−0.27, −0.04], $n_{obs} = 477$
TMB
60
40
20
0
$\widehat{\mu}_{median} = 4.00$
$\widehat{\mu}_{median} = 5.00$
high
(n = 121)
low
(n = 356)
group
